# Peritoneal Dialysis Fluid Supplementation with Alanyl-Glutamine Attenuates Conventional Dialysis Fluid-Mediated Endothelial Cell Injury by Restoring Perturbed Cytoprotective Responses

**DOI:** 10.3390/biom10121678

**Published:** 2020-12-15

**Authors:** Rebecca Herzog, Maria Bartosova, Silvia Tarantino, Anja Wagner, Markus Unterwurzacher, Juan Manuel Sacnun, Anton M. Lichtenauer, Lilian Kuster, Betti Schaefer, Seth L. Alper, Christoph Aufricht, Claus Peter Schmitt, Klaus Kratochwill

**Affiliations:** 1 Division of Pediatric Nephrology and Gastroenterology, Department of Pediatrics and Adolescent Medicine, Comprehensive Center for Pediatrics, Medical University of Vienna, 1090 Vienna, Austria; rebecca.herzog@meduniwien.ac.at (R.H.); silvia.tarantino@meduniwien.ac.at (S.T.); n11927077@students.meduniwien.ac.at (J.M.S.); anton_michael_lichtenauer@gmx.at (A.M.L.); lilian.kuster@gmx.at (L.K.); christoph.aufricht@meduniwien.ac.at (C.A.); 2 Christian Doppler Laboratory for Molecular Stress Research in Peritoneal Dialysis, Medical University of Vienna, 1090 Vienna, Austria; anja.wagner@meduniwien.ac.at (A.W.); markus.unterwurzacher@meduniwien.ac.at (M.U.); 3 Center for Pediatric and Adolescent Medicine, University of Heidelberg, 69120 Heidelberg, Germany; Maria.Bartosova@med.uni-heidelberg.de (M.B.); betti.schaefer@med.uni-heidelberg.de (B.S.); ClausPeter.Schmitt@med.uni-heidelberg.de (C.P.S.); 4 Zytoprotec GmbH, 1090 Vienna, Austria; 5 Division of Nephrology and Center for Vascular Biology Research, Beth Israel Deaconess Medical Center, Boston, MA 02215, USA; salper@bidmc.harvard.edu; 6 Department of Medicine, Harvard Medical School, Boston, MA 02115, USA

**Keywords:** peritoneal dialysis, vascular damage, l-Alanyl-l-glutamine, vasculopathy

## Abstract

Long-term clinical outcome of peritoneal dialysis (PD) depends on adequate removal of small solutes and water. The peritoneal endothelium represents the key barrier and peritoneal transport dysfunction is associated with vascular changes. Alanyl-glutamine (AlaGln) has been shown to counteract PD-induced deteriorations but the effect on vascular changes has not yet been elucidated. Using multiplexed proteomic and bioinformatic analyses we investigated the molecular mechanisms of vascular pathology in-vitro (primary human umbilical vein endothelial cells, HUVEC) and ex-vivo (arterioles of patients undergoing PD) following exposure to PD-fluid. An overlap of 1813 proteins (40%) of over 3100 proteins was identified in both sample types. PD-fluid treatment significantly altered 378 in endothelial cells and 192 in arterioles. The HUVEC proteome resembles the arteriolar proteome with expected sample specific differences of mainly immune system processes only present in arterioles and extracellular region proteins primarily found in HUVEC. AlaGln-addition to PD-fluid revealed 359 differentially abundant proteins and restored the molecular process landscape altered by PD fluid. This study provides evidence on validity and inherent limitations of studying endothelial pathomechanisms in-vitro compared to vascular ex-vivo findings. AlaGln could reduce PD-associated vasculopathy by reducing endothelial cellular damage, restoring perturbed abundances of pathologically important proteins and enriching protective processes.

## 1. Introduction

Peritoneal dialysis (PD) is a home-based, renal replacement therapy used by 300,000 end stage renal disease patients worldwide. As an alternative to hemodialysis, PD offers advantages in cost, quality of life and early survival [1]. The hyperosmolar PD fluid fills and is drained from the peritoneal cavity several times per day, promoting removal of water and solutes from the blood into the PD fluid during each intraperitoneal dwell period. Long-term clinical outcome of PD is strongly associated with the ability of this therapy to provide adequate removal of small solutes and fluid by continuous transport and ultrafiltration via the peritoneal membrane [2,3]. However, although conventional PD fluids are effective in removing excess water and waste products from uremic patients, they are bioincompatible in their high concentrations of glucose and its degradation products, their low pH, and their lactate buffer system [4]. 

Loss of ultrafiltration capacity in PD patients is often associated with peritoneal vascular changes, manifest as diabetes-like vasculopathy and angiogenesis [5,6,7]. The endothelial monolayer lining the vessel lumen controls vessel barrier function and thereby influences peritoneal substrate transport and ultrafiltration. According to the “3-Pore-Model”, the peritoneal vascular endothelium is considered the primary transport barrier in PD defining membrane function, whereas submesothelial fibrosis only impacts fluid and solute kinetics in severe cases [8,9,10]. It also influences peritoneal inflammation and host defenses by allowing translocation of immune cells into the peritoneal cavity [11,12]. Despite the association between ultrafiltration failure and peritoneal vascular changes, little is known about the effect of PD fluids on endothelial cell function and the pathophysiological mechanisms involved.

The dipeptide alanyl-glutamine (AlaGln) has recently shown cytoprotective effects when added to PD fluid, including preservation of mesothelial cells in vitro [13] and attenuation of the exuberant angiogenesis seen in long-term rodent models of PD [14]. These effects have been reflected in early phase clinical trials as restoration of effluent cell stress responses and improved biomarkers of peritoneal health. In a randomized clinical phase II trial, eight weeks treatment with AlaGln in PD fluid decreased peritoneal protein loss [15,16]. This clinically important effect might be explained by preserved peritoneal membrane and vessel integrity, but the role of AlaGln in preservation of peritoneal endothelial cell function remains unclear.

In this study, we characterized the response of endothelial cells to conventional PD fluids in vitro using the well-established cell model of human umbilical vein endothelial cells (HUVEC). To assess the validity of this approach, we compared these findings to omental arterioles isolated from peritoneal biopsies of children undergoing PD with the same PD fluid. To this end, we compared comprehensively the molecular mechanisms of endothelial cell pathology in vitro to arteriolar pathomechanisms active in vivo, i.e., reflecting vascular pathology, by proteomic and bioinformatic analysis, integrating current understanding of PD-related cellular injury and stress responses [17,18,19,20]. We then evaluated the effect of AlaGln addition to conventional PD fluid on these parameters in HUVEC in vitro. This strategy allowed characterization of endothelial cell injury and stress responses during exposure to PD fluid, and their modulation by added AlaGln as a promising therapeutic approach to counteract PD induced pathomechanisms of vasculopathy.

## 2. Materials and Methods

Standard chemicals were from Sigma–Aldrich (St Louis, MO, USA) unless otherwise specified. Cell culture plastics were from TPP-Techno Plastics Products AG, Trasadigen, Switzerland.

### 2.1. Cell Culture

Primary human umbilical vein endothelial cells (HUVECs, Lonza, Basel, Switzerland) were cultured in 25 cm^2^ and 75 cm^2^ tissue culture flasks in endothelial basal medium (EBM-2, Lonza, Basel, Switzerland) containing 2% fetal calf serum (FCS) supplemented with endothelial cell growth supplements (EGM-2; Lonza) in humidified 5% CO_2_ at 37 °C. For experiments, cells from passages 2-5 were grown on 12-well plates.

### 2.2. Experimental PD Fluid Exposure Setting

HUVECs were exposed to experimental solutions for up to 24 h. All test fluids were sterile-filtered before usage. Each experiment consisted of three independent samples in biological replicates on separate culture plates. For PD fluid incubation, cells were first exposed for 1 h to pure glucose-based PD fluid (Dianeal PD4 3.86% glucose, Baxter, Castlebar, Ireland), or to the same PD fluid supplemented with AlaGln dipeptide (8 mM, Dipeptiven; Fresenius Kabi, Bad Homburg, Germany), or to normal medium without growth factors as control. Cells were subsequently exposed for 24 h to the above solutions diluted 1:1 with culture medium and brought to 2% FCS. Cells were then washed three times (250 mM sucrose, 10 mM Tris/HCl, pH 7) and lysed in 125 µL per well of lysis buffer (30 mM Tris, pH 8.5, 7 M urea, 2 M thiourea, 4% CHAPS, 1 mM EDTA, one tablet of Complete Protease Inhibitor (Roche, Basel; Switzerland) per 100 mL, and one tablet of PhosStop Protease Inhibitor (Roche) per 100 mL). Total protein concentration was determined with the 2D-Quant kit (GE Healthcare, Uppsala, Sweden) per manufacturer’s manual. Lysates were stored at −80 °C until further processing. Each experiment was repeated three times with cells from different donors.

### 2.3. Arterioles

9 omental biopsies from the pediatric biopsy biobank (mean age 5.7 years) were included in this study. PD patients were treated with conventional PD fluid (mean PD vintage 12.5 months). Age-matched controls (*n* = 5) with normal renal function and no history of peritonitis undergoing elective laparoscopic fundoplication were also included. In patients on PD, the biopsy sampling site was at least 5 cm away from the PD catheter entry site (reasons for surgery: catheter exchange due to dysfunction or abdominal surgery for renal transplantation). Written informed consent was obtained from parents, and from patients as appropriate. The study was performed according to the Declaration of Helsinki and registered at www.clinicaltrials.gov (NCT01893710). The study was part of the International Pediatric Dialysis Network (IPDN; www.pedpd.org). Patients with a BMI of >35 kg/m^2^ and with chronic inflammatory diseases were excluded.

Specimens obtained during surgery were instantaneously frozen with liquid nitrogen and stored at −80 °C. Arterioles macroscopically located within fat tissue and microscopically at least 1mm distant from the peritoneal surface were micro-dissected. Elastica van Gieson stainings of the arteriolar elastic lamina were used as templates for microdissection of arterioles from cresol violet–stained neighboring sections. 100 µm thick tissue slices were cut with a cryotome and 30 deep-frozen arteriolar rings per patient were micro-dissected using a stereo microscope and a 27 gauge needle. Arteriolar rings were lysed in 100 µL lysis buffer (see above). The resulting lysates were stored at −80 °C until further processing. Quality control and adjustment of protein loading for trypsin digestion was performed by SDS-PAGE.

### 2.4. Cell Damage Assay

Cell damage following treatments of cells was assessed from lactate dehydrogenase (LDH) release into cell culture supernatants (TOX-7 LDH Kit, Sigma) per manufacturers’ instructions.

### 2.5. Immunofluorescence

Cells were cultured to confluence and treated with PD fluid without or with 8 mM AlaGln. HUVECs were fixed in ethanol at −20 °C for 5 min, permeabilized (0.5% TritonX-100 in PBS) and blocked (5% bovine serum albumin in PBS) for one hour at room temperature. Incubation with the primary antibody (zonula occludens-1 (ZO-1), Thermo Fisher Scientific, Waltham, MA, USA) was performed overnight at 4 °C. Secondary, fluorophore-labeled antibody was added for one hour. Nuclei were stained with DAPI, and cells were imaged with a laser-scanning confocal microscope (Leica TCS SP8, Wetzlar, Germany).

### 2.6. Viability Assay

To assess cell viability by neutral red uptake, cells were seeded on 96-well plates and exposed to different treatments (see above) and different periods of 1:1 dilutions following pure PDF exposures. Neutral red uptake measures amounts of incorporated stain in lysosomes and was performed using a standard reagent (Sigma-Aldrich) according to the manufacturer’s protocol.

### 2.7. Fluorescence Labeling of Proteins and 2-DE

An internal pooled standard (IPS) containing equal parts of each sample was used as standard in all gels. Protein labeling was accomplished using the Refraction-2D Labeling Kit (NH DyeAGNOSTICS, Halle, Germany) per manufacturers’ protocol. Samples were labeled with G300 reagent (0.2 nmol) whereas IPS was labeled with G200 reagent (0.2 nmol). Equal 40 µg amounts of IPS and sample were mixed and brought to a final volume of 450 µL with rehydration buffer (5 M urea, 0.5% CHAPS, 0.5% Pharmalyte and 12 µL/mL of DeStreak reagent (GE Healthcare, Chalfont St. Giles, UK). The mixture was applied to one IPG strip (ReadyStrip pH 3-10, non-linear, 24 cm, Bio-Rad, Hercules, CA, USA), which was covered with mineral oil (Bio-Rad) before active rehydration at 50 V for 12 h at 20 °C using a Protean IEF Cell (BioRad, Hercules, CA, USA). Isoelectric focusing (IEF) was run by constantly increasing the voltage to 8000 V, applying in total 84 kVh using a current limit of 30 µA per strip. After focusing, strips were stored at −80 °C until further use. Before running the second dimension, each strip was incubated for consecutive 15 min periods in 2 mL equilibration buffer (6 M urea, 2% (*w*/*v*) SDS, 25% (*w*/*v*) glycerol, and 3.3% (*v*/*v*) 50 mM Tris/HCl pH 8.8, bromophenol blue) first supplemented with 20 mg of dithiothreitol (DTT) and then with 96 mg of 2-iodoacetamide (IAA). Subsequent SDS–PAGE used lab-cast gels (12% vol/vol acrylamide) and a Dodeca Plus cell (Bio-Rad) at 20 °C with a first phase current of 60 mA and second phase current of 200 mA until the tracking dye reached the bottom of the gel.

Image Analysis: Gels were washed with deionized water and scanned using a Typhoon Trio laser scanner (GE Healthcare) at the excitation and emission wavelengths as described in the labeling kit manual (G-Dye200: ex/em 554/575 nm; G-Dye300: ex/em 648/663 nm). Gel images were analyzed using Delta2D 4.3 software (Decodon GmbH, Greifswald, Germany) with group-wise image alignment and spot detection on the resulting fused image.

### 2.8. CBB Staining and In-Gel Digestion

For spot identification, preparative gels with 400 µg of unlabeled IPS were separated in 2-DE as previously described and gels were stained with Coomassie Brilliant Blue (CBB). Protein spots were fixed overnight (10% acetic acid, 40% ethanol) followed by 35 min water washes. After overnight incubation with CBB staining solution (8% (*w*/*v*) ammonium sulfate, 2% (*w*/*v*) orthophosphoric acid (85%), 20% (*v*/*v*) methanol and 1% (*v*/*v*) CBB stock solution (2.5% (*w*/*v*) Coomassie Brilliant Blue G250 dissolved in H_2_O)) the staining was intensified by 30 min incubation in 20% ammonium sulfate (in H_2_O), then briefly destained with 10% glycerol, 20% methanol (in H2O). All incubation steps were carried out on a horizontal rotary shaker. Spots were excised with the EXQuest Spot Cutter (BioRad) and subjected to tryptic in-gel digestion. Excised gel plugs were washed (100 mM NH_4_HCO_3_, 100 mM NH_4_HCO_3_/ethanol (1:1) and acetonitrile) until destained. After reduction (10 mM DTT in 25 mM NH_4_HCO_3_) for 1 h at 56 °C, the samples were alkylated (55 mM IAA in 25 mM NH_4_HCO_3_) for 45 min at room temperature in the dark. Following washing steps (100 mM NH_4_HCO_3_ and acetonitrile) samples were digested with 0.39 µg trypsin in 50mM NH_4_HCO_3_ overnight at 37 °C. The cleaved peptides were eluted from the gel plugs with sonication in acetonitrile/H_2_O/trifluoroacetic acid (TFA) (50:45:5). Eluates were dried by vacuum centrifugation (Concentrator plus, Eppendorf, Hamburg, Germany) and the peptides were redissolved with 0.1% TFA and desalted with C18-ZipTip columns (Millipore; Billerica, MA, USA) per manual. Briefly, the column matrix was wetted with acetonitrile and equilibrated with 0.1% TFA. Peptides were loaded onto the column, followed by washing with 0.1% TFA and direct elution onto two spots of the MALDI target (Thermo Fisher Scientific, Bremen, Germany) with α-cyano-4-hydroxycinnamic acid (10 mg/mL; CHCA; Fluka) in acetonitrile/0.1% TFA.

### 2.9. MALDI and Database Search

Mass spectrometric (MS) analyses were performed on a Matrix-assisted laser desorption ionization (MALDI) LTQ Orbitrap XL mass spectrometer (Thermo Fisher, Bremen, Germany). The instrument was operated in positive mode. MS spectra were acquired for a mass range from *m*/*z* 600–4000 with a resolution setting of 100,000 at *m*/*z* 400. Acquisition parameters were: automated spectrum filter (ASF) off, automated gain control (AGC) on, crystal positioning system (CPS) on, 5 scans/step. For tandem mass spectrometry (MS/MS) the mass spectrometer was operated in a data-dependent mode to switch between Orbitrap MS and LTQ MS/MS analyses. Parameters included precursor ion isolation in the linear ion trap, 3 mass units isolation width, 3 normalized collision energies (CID) 30%, 35%, 40%, activation q 0.25. The 10 most prominent ions were sequentially isolated for CID fragmentation in the linear ion trap. Spots that differed significantly between PD fluid and AlaGln-supplemented PD fluid were identified by LC MS/MS using a QqTOF compact (Bruker Daltonics, Billerica, MA) [21]. The acquired raw MS data files were processed (Mascot Distiller 2.7.1.0; Matrix Science, London, UK) and searched against the human SwissProt database (2014-10) using Mascot. Additional search parameters were: enzyme: trypsin/P; allowed missed cleavages: 2; fixed modifications: carbamidomethyl (C); variable modifications: oxidation (M); peptide tolerance: 5 ppm; MS/MS tolerance: 0.8 Da; peptide charge: 1+, 2+ and 3+. The ions score was set to 20 and standard scoring was used.

### 2.10. FASP and TMT Labeling of Human Omental Arterioles

Filter-aided sample preparation (FASP) was performed using 30 kDa molecular weight cut-off filters (Millipore). After reduction (83 mM DTT, 5 min at 99 °C), 30 µg of each sample was mixed with 200 µL UA buffer (8 M urea in 100 mM Tris-HCl, pH 8.5) in the filter unit and centrifuged (14,000× *g*, 15 min, 20 °C). Before alkylation (100 µL 55 mM IAA, 30 min at RT), proteins were washed again with UA buffer. Afterwards, proteins were washed 3 more times with 100 µL UA buffer and 100 µL 50 mM triethylammonium bicarbonate buffer (TEAB; 1 M, pH 8.5), respectively. Digestion was performed with trypsin in 50 mM TEAB (1:50 protease:protein ratio, Promega, Madison, WI, USA) at 37 °C overnight. Peptides were recovered with 40 µL 50 mM TEAB and 50 µL 0.5 M NaCl. After collection peptides were desalted using C18 microspin columns (5−60 μg, The Nest Group, Southborough, MA, USA), dried in a vacuum concentrator and reconstituted in 100 mM TEAB, pH 8.5 (Fluka). Labeling with TMT 10plex (Thermo Fisher Scientific, Waltham, MA, USA) was performed according to the instructions provided by the manufacturer. TMT reagents were reconstituted with acetonitrile and each sample was labeled with 1 vial of TMT reagent (800 µg). After incubation for 1h at RT the reaction was quenched by addition of 5% hydroxylamine (Sigma) in TEAB followed by incubation for 15 min at RT. Pooled samples were concentrated and desalted with C18 macrospin columns (30−300 μg, The Nest Group). Eluates were dried in a vacuum concentrator and reconstituted in 20 mM ammonia formate buffer, pH 10 before fractionation at basic pH. Two-dimensional liquid chromatography was performed by reverse-phase chromatography at high and low pH. In the first dimension peptides were separated on a Gemini-NX C18 (150 × 2 mm, 3 µm, 110 A, Phenomenex, Torrance, USA) in 20 mM ammonia formate buffer, pH 10, and eluted over 45 min by 5–70% acetonitrile gradient at 100 µL/min using an Agilent 1200 HPLC system (Agilent Biotechnologies, Palo, Alto, CA, USA). Ninety-six time-based fractions were collected and pooled into 12 HPLC vials. Organic solvent was removed in a vacuum concentrator and samples were reconstituted in 5% formic acid (similar to Bennett et al., J Proteomics 2011). Fractions were analyzed at low pH on an Ultimate 3000 RSLC nano coupled directly to an Orbitrap Fusion Lumos Tribrid mass spectrometer (both Thermo Fisher Scientific). Samples were injected into a reversed-phase C18 column (50 cm × 75 µm i.d., packed in-house) and eluted with a gradient of 6% to 65% mobile phase B over 94 min by applying a flow rate of 230 nL/min. MS scans were performed in the range from *m*/*z* 375–1650 at a resolution of 120,000 (at *m*/*z* = 200). MS/MS scans were performed choosing a top 15 method for peptide identification and relative quantification of TMT reporter ions with these parameters: resolution 50,000; normalized collision energy 38%; isolation width 0.5 *m*/*z*; dynamic exclusion 90 s.

### 2.11. SP3 and TMT Labeling of HUVEC

70 µg of each sample (medium, PD4 and PD4+AG) and an internal pooled standard (IPS) consisting of equal parts of all samples were used. Digestion was performed using single-pot, solid-phase enhanced sample preparation (SP3). Briefly, the reduced (10 mM DTT for 1 h at 56 °C) and alkylated (55 mM IAA, 30 min at RT) proteins were bound to SP3 beads (10:1 beads:protein ratio, GE Healthcare), washed with 80% ethanol and acetonitrile, and subjected to on-bead digestion with trypsin (1:50 protease:protein ratio, Promega) overnight at 37 °C in 50 mM ammonium bicarbonate, pH 8.5 (Sigma). After elution peptides were desalted using C18 macrospin columns (30−300 μg, The Nest Group), dried in a vacuum concentrator and reconstituted in 100 mM TEAB, pH 8.5 (Fluka). Labeling with TMT 10plex (Thermo Fisher Scientific) was performed according to the instructions provided by the manufacturer. TMT reagents were reconstituted with acetonitrile and each sample was labeled with 1 vial of TMT reagent (800 µg). After incubation for 1h at RT the reaction was quenched by addition of 5% hydroxylamine (Sigma) in TEAB and further incubation for 15 min at RT. Eluates were dried in a vacuum concentrator and reconstituted in 20 mM ammonia formate buffer, pH 10, before fractionation at basic pH. Two-dimensional liquid chromatography was performed by reverse-phase chromatography at high and low pH. In the first dimension peptides were separated on a Gemini-NX C18 (150 × 2mm, 3 µm, 110 A, Phenomenex, Torrance, USA) in 20 mM ammonia formate buffer, pH 10 and eluted over a 32 min gradient from 0% to 30% solvent B, followed by 6 min at 100% solvent B at 50 µL/min using an Ultimate 3000 RSLC micro system (Thermo Fisher Scientific) equipped with a fraction collector. Fractions were collected every 30 s to a total of thirty-six fractions. Organic solvent was removed in a vacuum concentrator and samples were reconstituted in 5% formic acid. Fractions were analyzed at low pH on an Ultimate 3000 RSLC nano coupled directly to a Q Exactive mass spectrometer (both Thermo Fisher Scientific). Samples were injected into a reversed-phase C18 column (50 cm × 75 µm i.d., packed in-house) and eluted with a gradient of 6% to 65% mobile phase B over 94 min by applying a flow rate of 230 nL/min. MS scans were performed in the range from *m*/*z* 375–1650 at a resolution of 70,000 (at *m*/*z* = 200). MS/MS scans were performed choosing a top 10 method for peptide identification and relative quantitation of TMT reporter ions with these parameters: resolution 35,000; normalized collision energy 33%; isolation width 1.2 *m*/*z*; dynamic exclusion 90 s.

### 2.12. Mass Spectrometry Data Analysis

The acquired raw MS data files were processed and analyzed using ProteomeDiscoverer (v2.2.0.388, Thermo Fisher). SequestHT was used as search engine and following parameters were chosen: database: Homo sapiens (SwissProt, https://www.uniprot.org/, 2019-03-06); enzyme: trypsin; max. missed cleavage sites: 2; static modifications: TMT6plex (K) and carbamidomethyl (C); dynamic modifications: oxidation (M), TMT6plex (peptide N-terminus) and acetyl (protein N-terminus); precursor mass tolerance: 10 ppm; fragment mass tolerance: 0.02 Da. For reporter ion quantitation the most intense *m*/*z* in a 20 ppm window around the theoretical *m*/*z* was used. Correction of isotopic impurities for reporter ion intensities was applied. Only unique peptides were used for quantitation, which was based on S/N values with an average S/N threshold of 10. Normalization was based on total peptide amount and scaling mode on all averages or control averages when an internal standard was used. Only peptides and proteins with FDR < 0.01 are reported. Single peptide IDs were excluded from the dataset.

### 2.13. Enrichment Map Analysis

Functional analysis of differentially abundant proteins was performed using the Database for Annotation, Visualization and Integrated Discovery (DAVID) (version 6.7). We used DAVID functional annotation chart tool which provides enrichment analysis for the identification of the over-represented biological terms by a gene list. Annotation databases included in the analysis were GO Biological Process (BP); GO molecular function (MF) and GO cellular component (CC). DAVID functional annotation chart analysis was loaded into Enrichment Map (www.baderlab.org/Software/EnrichmentMap), a Cytoscape plugin that allows visualization of large gene-sets in a concise network [22]. In the map, nodes represent significant enriched gene-sets (p≤0.05 corrected for multiple testing by the Benjamini-Hochberg (BH) method) while edges represent shared genes between two nodes. Node size represents the number of genes within the gene-set, while edge size represents the number shared genes between two nodes. Node color intensity ranges from red (*p* = 5 × 10^−8^) to white (*p* = 0.05), reflecting significance of enrichment. Map layout optimization was accomplished by removal of redundant nodes sharing the same genes and by highlighting prevalent biological functions (circled and labeled). Linked gene sets are those with overlap coefficient ≥ 0.5 (50% or more genes shared between gene sets).

### 2.14. Statistical Analysis

Statistical analyses and graphical representations of results were performed using Statistical Package for the Social Sciences 17 (SPSS Inc., Chicago, IL, USA), R (v3.5.1; http://www.r-project.org/), Prism 7 and 8 (GraphPad, La Jolla, CA, USA) and Venn Diagram Plotter (version 1.5.5228.29250). For LDH release results are expressed as mean ± standard error, normalized by sample protein concentration and compared using ANOVA and post-hoc Tukey’s test. *p*-values < 0.05 were considered significant. Proteomic spot quantification was based on IPS normalized spot volume (% volume) as exported from the quantitation table of Delta2D software. Mean spot volumes and relative coefficient variation (CV) together with significance values derived from group comparisons utilizing Student’s t-test, are listed in Supplemental Table S8. In DAVID enrichment analysis, enrichment p-values were corrected for multiple testing by the BH method and terms were considered enriched when *p*-values <0.05. Ingenuity Pathway Analysis (IPA 7.0, Qiagen, http//www.ingenuity.com) identified pathways and predicted up/down regulation patterns significantly affected by differentially abundant proteins, calculating a p-value for each functional pathway using a one-tailed Fisher exact test. Pathways with *p*-values < 0.05, after correction for multiple hypothesis testing with the BH procedure, were considered significantly enriched. The IPA z-score assessed the match of observed and predicted up/down regulation patterns and served as a predictor for the activation state.

### 2.15. Data Availability

Mass spectrometry data have been deposited into the ProteomeXchange Consortium (http://proteomecentral.proteomexchange.org) via the PRIDE partner repository with dataset identifiers PXD022170 and PXD022183.

## 3. Results

**Figure 1:**
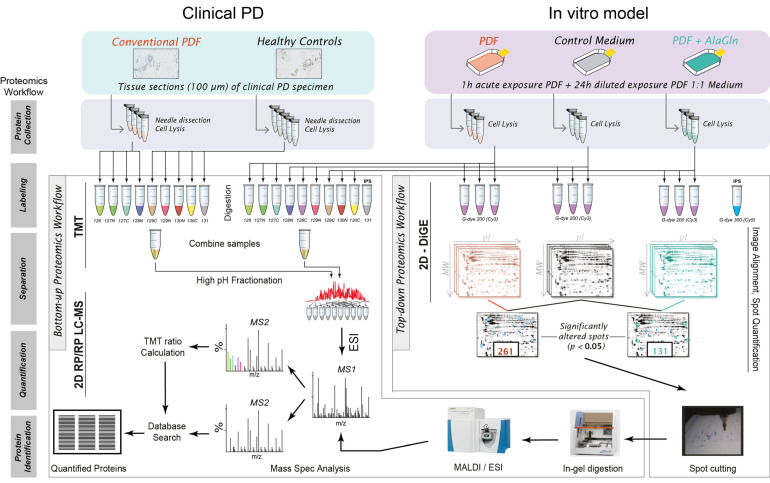
Experimental and analytical workflow. (**left**): Clinical PD samples (human omental biopsies) of pediatric PD patients and healthy controls; (**right**): in vitro samples from three different primary HUVEC cultures exposed to diluted conventional PD fluid (PDF) containing or lacking 8 mM alanyl-glutamine (AlaGln), or to control cell culture medium. TMT: tandem mass tags; ESI: electrospray ionization; MALDI: matrix-assisted laser desorption/ionization; IPS: internal pooled standard; 2D RP/RP LC-MS: two-dimensional reversed phase/reversed phase liquid chromatography tandem mass spectrometry; 2D-DiGE: two-dimension difference gel electrophoresis.

This systematic investigation of the proteomes of endothelial cells exposed to conventional and AlaGln-supplemented PD fluids was performed on two types of endothelial cell samples in a multiplex-approach based on tandem mass tags (TMT), off-line fractionation of digested proteins and shotgun (“bottom-up”) proteomics analysis, using orbitrap-type high resolution mass spectrometers. This proteome analysis was further complemented by a gel-based (“top-down”) proteomics approach (Figure 1).

### 3.1. Effect of Conventional PD Fluid on HUVEC

Primary human umbilical vein endothelial cells were stimulated using an in vitro test system with PD fluid. Normal cell culture medium served as physiological control. Using a high-performance proteomics approach, 3215 proteins were identified (Supplemental Table S1).

Quantitative proteomic analysis of arterioles isolated from peritoneal biopsies of children dialyzed with conventional PD fluid (*n* = 4) or healthy control donors undergoing elective surgery for a reason unrelated to either the kidney or dialysis (*n* = 5) identified and quantified 3128 proteins, very similar in number to in vitro proteome analysis, demonstrating excellent sensitivity of the approach in these tiny, infrequently available specimens (Supplemental Table S2).

**Figure 2:**
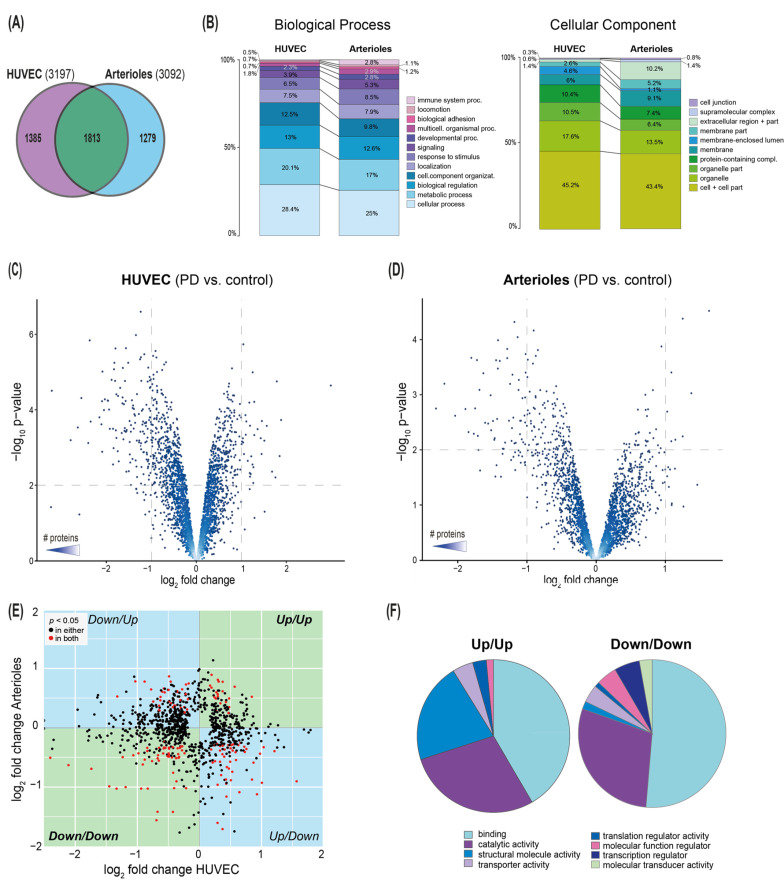
Proteomic differences of endothelial cells following PD exposure in vitro and peritoneal arterioles of PD patients. (**A**) Venn diagram illustrating the overlap between identified proteins in HUVECs and in arterioles (both TMT labeled/Bottom-up approach). (**B**) Bar charts of distribution of gene ontology (GO) biological process (left) and cellular component (right) associations to protein identifications in HUVECs and in arterioles, (not shown are these GO categories represented at levels <0.5%: reproductive process, reproduction, multi-organism process, cell population proliferation, biological phase, growth, behavior, biomineralization, rhythmic process, synapse, synapse part). (**C**) and (**D**) Volcano plots for regulated proteins (**C**) in HUVECs (exposed to PD fluid or to control cell culture medium) and (**D**) arterioles (PD patients vs. healthy controls). *p* < 0.01 for all points above dashed line. Color gradient indicates number of proteins. (**E**) Co-regulation analysis of proteins significantly regulated in either comparison (black) or in both comparisons (red): (comparisons: PD fluid-exposed vs. control cell culture medium-exposed HUVECs and PD fluid-exposed vs. healthy control arterioles. (**F**) Pie charts of distribution of gene ontology (GO) molecular function of proteins up-regulated or down-regulated in both comparisons.

Comparison of identified proteins (Figure 2A) of HUVEC and arterioles revealed 1813 or 40% of the 4477 identified proteins (corresponding to unique genes only; 1741 or 38% of the overall 4602 proteins) were shared between the two sample types. 1474 proteins (corresponding to 1385 unique genes) were present exclusively in HUVEC and 1387 proteins (1279 unique genes) exclusively in arterioles. Considering that two shotgun proteomics experiments of the same sample reach only 70–80% overlap ([23]; reflecting data-dependent acquisition strategy of the MS method) the degree of overlap is higher than expected from two distinct sample types.

We hypothesized that similarities and differences in the samples might be better represented on the level of gene ontology, as different proteins from the same category should have a higher chance of being detected in independent LC-MS experiments, provided similarity of underlying population. Indeed, the HUVEC proteome very much resembles the arteriolar proteome with a few noteworthy exceptions. Immune system processes observed in the clinical specimen are nearly undetectable in the in vitro endothelial cell culture system (Figure 2B). Similarly, proteins associated with the extracellular region are more evident in the arterioles than in primary HUVEC.

Comparison between HUVEC exposed to PD fluid or to control cell culture medium yielded 920 significantly altered proteins (*p* < 0.01), reduced by Benjamini-Hochberg (BH) (BH *p* < 0.01) multiple testing correction to 378 proteins (Supplemental Table S3) with most of these proteins downregulated in the PD group (Figure 2C). Among the most prominent upregulated proteins was endothelial cell specific molecule 1 (ESM1).

Comparison of arteriolar proteomes from conventional PD fluid-treated and healthy control biopsies revealed 192 proteins of significant differential abundance (*p* < 0.01; Supplemental Table S4), again, proteins downregulated following PD fluid exposure exceeded those upregulated (Figure 2D). We observed good coverage of the few upregulated proteins previously reported as effluent biomarkers of long-term PD, including retinol binding protein 4 (RBP4), alpha-1-microglobulin (AMBP) and apolipoprotein A4 (APOA4), and periostin (POSTN).

Candidate sets of PD-influenced proteins from both HUVEC and arterioles were subjected to pathway analysis and enriched canonical pathways were intersected. Pathways enriched both in peritoneal arterioles after PD therapy with conventional PD fluid and in HUVEC exposed in vitro to the same PD fluid are shown in Table 1. The main overlapping “structural protein” pathways between the two sample types included “cytoskeletal processes” (actin cytoskeleton signaling, Rho and Ras signaling) and “cell junctional processes” (e.g., epithelial adherens junctions). Sample-specific pathways enriched in the proteomes of HUVEC and of arterioles were extracted. Proteins already covered in the overlapping pathways were removed and the ratio of sample-specific proteins to all regulated proteins in the pathways were calculated. Proteins identified specifically in HUVEC were associated with mitochondrial dysfunction, phagosome processes, ubiquitination, RAN signaling and certain biosynthetic pathways (Supplemental Table S5). Proteins identified specifically in arteriolar pathways were related to the coagulation system, acute phase and atherosclerosis signaling, and detoxification pathways (Supplemental Table S6).

We next analyzed those overlapping proteins regulated by PD fluid in both HUVEC and arteriolar samples. As proteins of significantly differential abundance in both experiments (highlighted in red in Figure 2E) constitute only a fraction of those significantly altered in either individual experiment, we found no global correlation between corresponding proteins of the two sample types. Among proteins upregulated in both HUVEC and arterioles, structural proteins were over-represented; transcription-associated molecular functions were found over-represented in proteins down-regulated in both sample types (Figure 2F).

### 3.2. Cytoprotective Effects of AlaGln in an In Vitro Model of PD Using HUVEC

**Figure 3:**
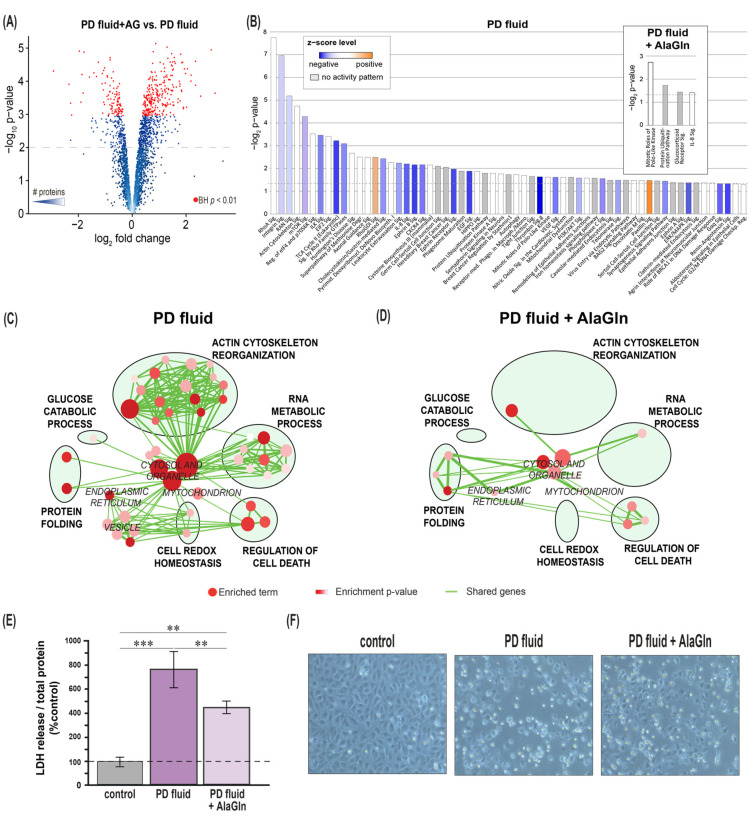
Alanyl-Glutamine (AlaGln) reduces PD fluid-induced molecular perturbations in and cell death of HUVEC. (**A**) Volcano plots for regulated proteins of PD vs. PD+AlaGln groups, *p* < 0.01 for all points above dashed line. Color gradient indicates number of proteins, red points: BH-corrected *p* < 0.05 (**B**) IPA canonical pathways significantly enriched following exposure to PD fluid or PD fluid with 8 mM alanyl-glutamine (top insert), passing a threshold of *p* < 0.05 and consisting of at least 3 different genes. The IPA activation z-score is a calculated prediction of activation or inhibition of regulators based on relationships with dataset genes and direction of change of dataset genes. It represents the bias in gene regulation that predicts whether the upstream regulator exists in an activated or inactivated state. (**C**,**D**) Biological process enrichment map: Maps of enriched GO terms in PD fluid-treated cells (**C**) and in AlaGln-supplemented PD fluid-treated cells (**D**) versus control. In the network, nodes represent enriched GO terms (*p*-value < 0.05) while edges represent shared genes between two nodes. Node size represents the number of genes within the enriched term and edge size represents the number of shared genes between two nodes. Color intensity node ranging from red (*p*-value = 5 × 10^−8^) to white (*p*-value = 5 × 10^−2^) reflects enrichment significance (BH-corrected for multiple testing). Prevalent biological functions were highlighted by circling and labeling clusters of functionally related terms. Gene Ontology Consortium terms represented enriched molecular function, cellular component and biological process. Map layout was optimized by removal of redundant nodes sharing the same genes. (**E**) Lactate dehydrogenase (LDH) release into cell supernatant, marker of cellular damage normalized to total cell protein, n = 3 individual experiments, each comprising 4 replicate samples, each measured in duplicate, *** *p* < 0.001, ** *p* < 0.01. (**F**) representative photomicrographs of cells exposed to test fluids, magnification 20×.

After demonstrating substantial overlap of the in vitro HUVEC model with ex vivo vascular findings, we compared HUVEC exposed to AlaGln-supplemented PD fluid to those exposed to PD fluid without AlaGln, for testing the potential of AlaGln to abrogate PD fluid-associated deleterious effects. The comparison revealed 359 differentially abundant proteins (BH-corrected *p* < 0.01, Figure 3A), many of which were upregulated by AlaGln-supplemented PD fluid as compared to conventional PD fluid. In contrast, comparison of AlaGln-supplemented PD fluid vs. control cell culture medium revealed only 85 differentially abundant proteins (BH corrected *p* < 0.01). This more “control-like” pattern is also reflected in fewer enriched and activated canonical pathways (n = 4) than evident in the comparison between conventional PD fluid and control cell culture medium (n = 62; Figure 3B, including only pathways with ≥3 proteins; Supplemental Table S7).

To broaden our understanding of AlaGln-mediated effects on endothelial cells, we also performed a gel-based proteome analysis focusing on intact proteins (“top-down” proteomics). The three HUVEC experiments generated a common spot pattern of 993 protein spots (Supplemental Figure S1a). 261 spots differed in abundance between PD fluid- and control cell culture medium-exposed HUVEC (*p* < 0.05). 131 spots differed in abundance between AlaGln-supplemented PD fluid and control cell culture medium (*p* < 0.05). Direct proteome comparison of cells exposed to conventional PD fluid versus AlaGln-supplemented PD fluid revealed 55 differentially abundant protein spots (*p* < 0.05). Successful protein identification in 182 spots (Supplemental Figure S1b) identified 114 unique proteins (Supplemental Table S8) and were used to define the molecular process landscape in HUVECs upon PD fluid exposure.

Enrichment map analysis of HUVEC exposed to PD fluid vs. control medium yielded 59 enriched GO terms, summarized in six main biological process clusters: glucose catabolic process, cell redox homeostasis, RNA metabolic process, protein folding, regulation of cell death, and actin cytoskeleton reorganization (Figure 3C, Supplemental Table S9). The largest cluster, actin cytoskeleton reorganization, contains the most highly enriched term of this map. PD fluid exposure perturbs the abundance of nuclear proteins found in complexes with RNA, regulating gene transcription, post-transcriptional modifications and elongation of protein translation. The cytotoxic insult from PD fluid exposure is reflected in altered cell redox homeostasis and enrichment of tightly interconnected proteins regulating cell death processes. The proteotoxic insult triggers chaperones in the protein folding cluster.

Supplementation with AlaGln markedly restored the molecular process landscape altered by PD fluid (Figure 3D, Supplemental Table S10). The remaining enriched clusters, “protein folding” (decrease to five biological processes) and “regulation of cell death” (increase to three) are selectively altered.

The effect of AlaGln supplementation during PD fluid exposure was also assessed by direct comparison, similar to the shotgun proteomics approach 48 of the 55 spots of differential abundance (*p* < 0.05) between HUVEC exposed to conventional PD fluid and AlaGln-supplemented PD fluid were identified (Supplemental Table S8) and annotated within the six main clusters perturbed by PD fluid exposure (Supplemental Table S11). Interestingly, 75.6% of these spots were restored or partially restored to control levels by AlaGln-supplemented PD fluid (Supplemental Figure S2).

Cytoprotection of endothelial cells by AlaGln addition to PD fluid was further investigated by assessment of LDH release into the cell culture supernatant. Whereas HUVEC exposure to PD fluid resulted in cellular injury as indicated by LDH increase of 765 ± 118% (*p* < 0.01 vs. medium control after 24 h), AlaGln supplementation significantly attenuated LDH release to 442 ± 38% (*p* < 0.05 vs. both PD fluid and medium control; Figure 3E). Addition of AlaGln also increased cell viability at shorter time points of 3 h and 6 h (Supplemental Figure S3). Change in cell morphology was consistent with the reduction in LDH release (Figure 3F), and cytoskeletal organization was restored with the addition of 8 mM AlaGln (Supplemental Figure S4).

## 4. Discussion

The vasculopathy evident in the peritoneal membranes of chronic PD patients resemble vascular complications of diabetes, and suggest shared endothelial pathomechanisms which lead to PD ultrafiltration failure [24]. We evaluated the suitability of an in vitro model of endothelial dysfunction and vascular complications (commonly used in diabetes research [25]) for investigation of the effects of PD fluids on injury and stress responses of endothelial cells. We therefore characterized changes in endothelial proteomes and stress responses following PD fluid-induced injury. The validity of this commonly used in vitro model is uncertain. We therefore compared the in vitro findings against arteriolar proteome findings. We then investigated the effects on the in vitro cell culture model proteome of PD fluid supplementation with AlaGln, an agent recently shown to attenuate angiogenesis in a rodent model of PD [14] and to reduce clinically relevant protein loss in an 8-week human cross-over trial [16].

Top-down proteomics techniques such as 2D-DiGE can detect processes regulated not merely by protein abundance but also/or by variations in protein activity, as they can detect both post-translational modifications and alternately spliced isoforms [26]. 2D-DiGE employs an internal standard in combination with fluorescent protein labels that allow quantification over a wider dynamic range [27] thereby allowing quantitative proteomics based on 2D gel electrophoresis [28]. However, gel-based proteomics methods are not as sensitive as gel-free methods, and protein identifications on 2D gels are limited by the intrinsic constraints of spot cutting, in-gel digestion, and peptide elution. Thus, gel-based proteomics, although providing valuable complementary information, was feasible only for the in vitro HUVEC model. State-of-the-art shotgun methods such as that used in our bottom-up proteomics workflow rely on tandem mass tag (TMT) multiplexing and high-resolution mass spectrometers and can identify and quantitate thousands of different proteins from microgram samples of total protein. One of the inherent limitations of the shotgun approach is the “bottom-up” inference of protein abundance from peptide abundance, often ignoring post-translationally modified or alternately spliced proteoforms. However, application of the LC-MS approach to our scarce arteriolar samples allowed deep proteome profiling for comparison with data generated from cell culture.

Considerable overlap in the proteomes from HUVEC and arteriolar samples as well as specific differences between different sample origins were evident. Patient arteriolar samples revealed a larger proportion of extracellular region components, reflecting greater prominence of extracellular matrix (ECM), secreted proteins and factors of intercellular communication, including plasma and interstitial proteins. Immune processes were also more prominent in arteriolar tissue than in HUVEC. Due to the fact that arterioles not exclusively consist of endothelial cells but also of vascular smooth muscle cells, pericytes and in disease states immune and EndMT cells [29], these findings are expected and a confirmation of the sensitivity of our proteomics approach. Nevertheless, the large overlap of identified proteins and regulated biological processes by PD fluid exposure of a cell monolayer system and a 3D tissue shows the ability of the in vitro system to model biologically relevant pathomechanisms.

Proteomic evaluation of cell responses to PD fluid in HUVEC revealed shared pathways associated mainly with cytoskeletal and cell junction signaling. In the top-down approach, 59 terms associated with differential protein abundance were integrated in six enrichment clusters. Interestingly, some of the most strongly upregulated arteriolar proteins, including RBP4, have been previously reported in PD literature, suggesting specific RBP4 enrichment in vasculature as well as in dialysis effluent of long-term PD patients [30,31]. Indeed, the largest cluster in the molecular landscape in both proteomic analyses comprised cellular processes associated with cytoskeletal re-arrangement, reflecting typical hallmarks of pathological changes in the peritoneal membrane, endothelial dysfunction, and angiogenesis. In the top-down approach, HUVEC exposure to PD fluid perturbed major regulators of actin dynamics such as cofilin-1 and F-actin-capping protein, and regulators of actin cytoskeletal structure such as the ERM (ezrin, radixin, moesin) protein family.

Many of the peritoneal changes in chronic PD have been attributed to pathological stresses imposed by non-physiological glucose-based PD fluids [32]. PD fluid hyperosmolarity is achieved by a high glucose concentration, readily explaining the enrichment of the “glucose catabolic process cluster” in this study. Similar to our findings in mesothelial cells [20], key enzymes of the glucose-catalytic pathway, such as the glycolytic enzymes aldolase (ALDOA) and α-enolase (ENOA) were consistently increased in abundance in both proteomic approaches (shotgun and top-down), whereas triose-phosphate isomerase 1 (TPI1) was slightly decreased in the top-down data. Glucose-6-phospate dehydrogenase (G6PD) and 6-phosphogluconolactonase (PGLS; identified only by the top-down method), the first two enzymes of the pentose phosphate pathway (PPP) were significantly altered in PD fluid-exposed HUVEC. In contrast to our findings in mesothelial cells, the PPP rate-limiting enzyme, G6PD, was decreased in PD-exposed HUVEC. High glucose leads to oxidative stress by inhibiting the PPP through depletion of NADPH levels and downregulation of the glutathione system [25]. In accordance with these observations, glutathione S-transferase (GSTP1) abundance was decreased in all three proteomic datasets (although significant only in the top-down experiment), emphasizing impairment of endothelial cell antioxidant defense by high glucose.

Peritoneal cells respond to PD fluid-induced stress by activating various cytoprotective cellular response mechanisms, reflected in our HUVEC dataset by a “protein folding” cluster. Protein folding is mediated by the heat shock protein (HSP) family of “chaperones”, which protect human peritoneal mesothelial cells from PD fluid-induced oxidative stress and mitochondrial injury [17,33]. HUVEC exposure to PD fluid increased abundance of HSP70 family members, including the cytoplasmic HSP70 (HSPA1), the ER chaperone GRP78 (HSPA5), and the mitochondrial chaperone mortalin (HSPA9). In diabetes, oxidative stress impairs the heat stress response and unfolded protein recovery [34]. We have shown in mesothelial cells that PD fluid exposure may also cause an inadequate cellular stress response, potentially mediated by GDP toxicity, leading to reduced stress resistance [13,19] and, indeed, HSPA1 was suppressed in the arterioles dataset.

The dynamic balance in HUVEC between PD fluid-mediated cytotoxic injury and counteracting stress responses is represented by the cluster “regulation of cell death”. Intermittent high glucose levels, comparable to those in PD, enhance apoptosis related to oxidative stress in HUVECs [35]. If high glucose stress overcomes cellular control of levels of misfolded proteins, the same protein disulfide isomerases (PDIs) or HSPs (such as HSP27 (HSPB1)) conferring cytoresistance and enhanced cellular repair can also predispose to apoptosis [36,37].

Well defined perturbations of molecular processes and pathways during stress may represent highly attractive therapeutic targets to counteract endothelial cell injury. Supplementation of PD fluid with AlaGln, a stable dipeptide of the conditionally essential amino acid glutamine, decreased cellular damage and restored cellular stress responses in an in vitro mesothelial cell PD model [13] by modulation of HSP abundance and protein *O*-GlcNAcylation [38]. PD fluid exposure models in uremic rats and mice exhibited reductions in peritoneal thickness and in markers of fibrosis and angiogenesis while the peritoneal surface proteome was restored [14,39]. These findings were recently transferred into the clinical setting, where AlaGln-treated patients showed increased peritoneal cell HSP expression [15], enhanced ex-vivo stimulated cytokine release from and attenuated markers of basal inflammation in peritoneal efflux cells [40], reduced protein loss [16], and reduced markers of oxidative stress in PD effluent [16,40,41].

In the current study, AlaGln restored endothelial cell processes in vitro through significant reduction of perturbation in protein abundance and improved endothelial cell survival. The data confirm recently reported cytoprotective effects of PD fluid supplementation with AlaGln in other models [13,14,15]. Reduced LDH release (a marker of cell membrane damage) as well as increased neutral red uptake (a marker of cell viability) corresponded to normalized cell morphology.

Our combined proteomic and bioinformatic analysis in HUVEC also demonstrated marked restoration of the molecular process landscape by AlaGln, reflecting abrogation of many PD-induced processes and almost complete resolution of all clusters except “regulation of cell death” and “protein folding”, with the latter enriched by a novel AlaGln-induced term “chaperone binding”.

Addition of AlaGln to PD fluid attenuated metabolic perturbation in glucose catabolic and cell redox homeostasis clusters by restoring abundance of high glucose-mediated oxidative stress regulators such as peroxiredoxin II (PRDX2), also a negative regulator of PDGF [42]. AlaGln addition also restored levels of the ER chaperones TXND5, PDIA3, and P4HB. Additionally identified was HSP27, the prototype small heat shock protein family member involved in multiple cytoprotective functions during cytoskeletal organization, protein degradation, cellular signaling mechanisms and prevention of apoptotic cell death [43]. Addition of AlaGln further restored HUVEC proteins regulating actin dynamics following glucose stress such as Cofilin 1 (CFL1), an actin severing protein, [44,45,46]. These, together with restoration of levels of ERM proteins, caldesmon (CALD1) [47] and vinculin (VCL), control cytoskeletal mechanics, stress fiber formation, cell spreading, and lamellipodia formation, important processes during early angiogenesis likely involved in endMT [48]. Proteomic results on cytoskeletal restoration were supported by immunostaining for a structural protein (ZO-1) representative of the cytoskeletal and junction-related processes enriched in the bioinformatic analysis. Vimentin (VIM), considered a hallmark of endothelial-mesenchymal transition [49], wound healing and endothelial sprouting in early angiogenesis [50] was also restored in abundance upon AlaGln supplementation of PD fluid, potentially reflecting the molecular mechanism of its reduction in peritoneal angiogenesis in a rat PD model [14].

Our data in HUVEC confirm previously reported pleiotropic effects of AlaGln on cellular metabolism, oxidative stress and cytoprotective responses, including restoration of the cellular stress proteome [13,51,52,53]. Our findings also agree with recent research suggesting metabolic perturbation in endothelial cells as a target for intervention in the setting of PD [25].

Limitations of the experiment with AlaGln addition include the lack of a control, such as equimolar amounts of a non-metabolizable di-peptide, or comparison to equimolar amounts of other amino acids for comparison. Those additional experimental conditions would increase the comprehensive understanding of (alanyl)-glutamine-mediated cytoprotection, but also the complexity, interpretation, and translation of findings from previous studies in mesothelial cells [13,38] or clinical trials of AlaGln in PD [15,16,40].

Although the molecular mechanisms by which AlaGln exerts its cytoprotective actions are not yet fully elucidated, this study corroborates recent data on endothelial specific peritoneal structural alterations, including cytoskeletal and cellular junction rearrangement as potential pathways influenced by AlaGln to maintain peritoneal barrier function [54].

## 5. Conclusions

Our study provides novel information on the value of experimental PD in vitro studies using HUVEC. Even though this model comes with several limitations, we were able to demonstrate a large overlap with the in vivo situation. Our studies further provide insights into biological processes and molecular perturbations at the proteome level in endothelial cells exposed to conventional PD fluid. Cellular stress responses in HUVEC exposed to PD fluids in vitro overlap with those observed in arteriolar samples from peritoneal biopsies of children undergoing PD with the same type of PD fluid. Our proteomic findings support recent literature on PD-associated pathomechanisms and cytoprotective actions of AlaGln and suggest potential molecular targets to reduce PD-associated damage. AlaGln reduces endothelial cellular damage, restores perturbed abundances of pathologically important proteins and enriches protective processes. Future, large scale comparative studies are needed to delineate detailed molecular mechanisms of CKD and specifically PD fluid components related vasculopathy. Transfer of our in vitro endothelial findings with AlaGln supplementation to clinical interventional settings, combined with detailed mechanistic studies linking the identified process clusters to clinical outcome, may enable to counteract the pathological side-effects of PD therapy in the peritoneal membrane and the local and systemic vascular system.

## Figures and Tables

**Table 1:** Pathways enriched both in peritoneal arterioles after PD therapy with conventional PD fluid and in HUVEC exposed in vitro to the same PD fluid.

Ingenuity Canonical Pathways	Arterioles *p*-Value	HUVEC *p*-Value	Arterioles *z*-Score	HUVEC *z*-Score	Arterioles Molecules	HUVEC Molecules
Actin Cytoskeleton Sig.	0.000 *	0.000 *	−1.265	0.000	KRAS, MYL6, PPP1R12A, MYLK, ARHGEF12, GRB2, ACTA2, MYL9, WASF2, CFL2, PPP1R12B	ACTB, ARHGAP35, ARHGEF1, ARPC2, BCAR1, CFL1, DOCK1, MAPK3, MYH10, MYL12B, PFN1, PFN2, ROCK1, SHC1
Agrin Interactions at Neuromuscular Junction	0.026	0.043			KRAS, CTTN, ACTA2	ACTB, AGRN, MAPK3, UTRN
Aldosterone Sig. in Epithelial Cells	0.017	0.048			HSPB1, KRAS, HSPA1A/HSPA1B, GRB2, DNAJB4	DNAJB1, DNAJB6, HSP90AB1, ITPR3, MAPK3, SLC12A2
Axonal Guidance Sig.	0.001 *	0.003			TUBB4A, MYL6, KRAS, GNAO1, TUBB3, ARHGEF12, GRB2, ABLIM1, MYL9, PPP3CA, ERAP2, CFL2	ACE, ARPC2, BCAR1, CFL1, DOCK1, EFNB2, GNAI2, LNPEP, MAPK3, MYL12B, NRP2, PFN1, PFN2, PLXNB2, ROCK1, SHC1, TUBG1
Breast Cancer Regulation by Stathmin1	0.002 *	0.019			TUBB4A, KRAS, ARHGEF17, TUBB3, PPP1R12A, ARHGEF12, GRB2	ARHGEF1, GNAI2, ITPR3, MAPK3, PPP2R1A, ROCK1, SHC1, TUBG1
CXCR4 Sig.	0.016	0.007	−2.000	−1.414	KRAS, MYL6, GNAO1, GRB2, MYL9	BCAR1, DOCK1, FNBP1, GNAI2, ITPR3, MAPK3, MYL12B, ROCK1
Clathrin-mediated Endocytosis Sig.	0.000 *	0.043			LYZ, APOA4, CTTN, GRB2, ACTA2, RBP4, PPP3CA, EPN1, ITGB4	ACTB, ARPC2, DAB2, ITGB5, PICALM, RPS27A, TFRC
EIF2 Sig.	0.014	0.000 *	0.000	0.000	KRAS, RPL7A, RPS9, GRB2, ACTA2, RPL27	ACTB, EIF3B, EIF3G, MAPK3, RPL18A, RPL3, RPS27A, RPS27L, RPS5, RPS8, RPSA, SHC1
ERK/MAPK Sig.	0.031	0.043	−1.342	−1.633	HSPB1, KRAS, CREB1, PPP1R12A, GRB2	BCAR1, DOCK1, MAPK3, PPP2R1A, SHC1, STAT1, YWHAB
Ephrin B Sig.	0.023 *	0.007		−2.000	GNAO1, ABI1, CFL2	CFL1, EFNB2, GNAI2, MAPK3, ROCK1
Ephrin Receptor Sig.	0.004 *	0.010	−2.449	−1.890	KRAS, CREB1, GNAO1, ABI1, GRB2, CFL2	ARPC2, BCAR1, CFL1, EFNB2, GNAI2, MAPK3, ROCK1, SHC1
Epithelial Adherens Junction Sig.	0.000 *	0.042			ZYX, TUBB4A, KRAS, MYL6, TUBB3, ACTA2, MYL9, EPN1	ACTB, ARPC2, CTNNA1, JUP, MYH10, TUBG1
Germ Cell-Sertoli Cell Junction Sig.	0.000 *	0.008 *			ZYX, TUBB4A, KRAS, TUBB3, GRB2, ACTA2, EPN1, CFL2	ACTB, BCAR1, CFL1, CTNNA1, FNBP1, JUP, MAPK3, TUBG1
ILK Sig.	0.000 *	0.000 *	−2.333	−1.265	VIM, MYL6, CREB1, TGFB1I1, PPP1R12A, GRB2, ACTA2, MYL9, ITGB4, CFL2, ILKAP	ACTB, CFL1, DOCK1, FLNB, FNBP1, ITGB5, LIMS1, MAPK3, MYH10, PPP2R1A, PTGS2
Integrin Sig.	0.000	0.000 *	−2.121	−0.500	ZYX, KRAS, CTTN, PPP1R12A, MYLK, GRB2, ACTA2, MYL9, ITGB4, PPP1R12B, ILKAP	ACTB, ARF3, ARF5, ARPC2, BCAR1, CAV1, DOCK1, FNBP1, ITGB5, LIMS1, MAPK3, MYL12B, PFN1, PFN2, ROCK1, SHC1, TSPAN6
Oncostatin M Sig.	0.046	0.034			KRAS, GRB2	MAPK3, SHC1, STAT1
Paxillin Sig.	0.020	0.034		1.000	KRAS, GRB2, ACTA2, ITGB4	ACTB, BCAR1, DOCK1, ITGB5, PTPN12
Phospholipase C Sig.	0.000 *	0.028	−2.111	−1.134	KRAS, MYL6, CREB1, ARHGEF17, PPP1R12A, ARHGEF12, GRB2, MYL9, PPP3CA, MARCKS, PPP1R12B	ARHGEF1, FNBP1, HDAC3, ITPR3, MAPK3, MARCKS, MYL12B, PLD3, SHC1
Protein Kinase A Sig.	0.002 *	0.016	0.816	0.000	MYL6, AKAP12, ADD1, CREB1, PPP1R12A, MYLK, LIPE, MYL9, PPP3CA, ADD3	AKAP12, AKAP9, FLNB, GNAI2, ITPR3, MAPK3, MYH10, MYL12B, PTGS2, PTPN12, PTPRB, ROCK1, YWHAB
Regulation of Actin-based Motility by Rho	0.000 *	0.000 *	0.000	0.378	MYL6, PPP1R12A, MYLK, ACTA2, MYL9, PPP1R12B	ACTB, ARPC2, CFL1, FNBP1, MYL12B, PFN1, PFN2, ROCK1
Remodeling of Epithelial Adherens Junctions	0.003 *	0.026			ZYX, TUBB4A, TUBB3, ACTA2	ACTB, ARPC2, CTNNA1, TUBG1
RhoA Sig.	0.000 *	0.000 *	−1.508	0.000	CDC42EP1, MYL6, CDC42EP4, PPP1R12A, MYLK, ARHGEF12, SEPT9, ACTA2, MYL9, CFL2, PPP1R12B	ACTB, ANLN, ARHGAP35, ARHGEF1, ARPC2, CFL1, CIT, MYL12B, NRP2, PFN1, PFN2, PKN1, ROCK1, SEPTIN5
RhoGDI Sig.	0.000 *	0.003	0.632	0.707	MYL6, ARHGEF17, GNAO1, PPP1R12A, ARHGEF12, ACTA2, MYL9, WASF2, PPP1R12C, CFL2, PPP1R12B	ACTB, ARHGAP35, ARHGEF1, ARPC2, CFL1, FNBP1, GNAI2, MYL12B, ROCK1
Sertoli Cell-Sertoli Cell Junction Sig.	0.000 *	0.035			SPTBN1, TUBB4A, KRAS, MAP3K20, TUBB3, GUCY1B1, PRKG1, ACTA2, EPN1	ACTB, BCAR1, CTNNA1, F11R, JUP, MAPK3, TUBG1
Sig. by Rho Family GTPases	0.000 *	0.001 *	−1.807	−1.265	MAP3K20, CDC42EP4, GNAO1, PPP1R12A, ARHGEF12, ACTA2, MYL9, PPP1R12C, CFL2, PPP1R12B, VIM, CDC42EP1, MYL6, ARHGEF17, MYLK, GRB2, SEPT9	ACTB, ARHGEF1, ARPC2, CFL1, CIT, FNBP1, GNAI2, MAPK3, MYL12B, PKN1, ROCK1, SEPTIN5
Thrombin Sig.	0.000 *	0.023	−2.000	−2.646	KRAS, MYL6, CREB1, GNAO1, PPP1R12A, MYLK, ARHGEF12, GRB2, MYL9, PPP1R12B	ARHGEF1, FNBP1, GNAI2, ITPR3, MAPK3, MYL12B, ROCK1, SHC1
Virus Entry via Endocytic Pathways	0.018	0.033			KRAS, GRB2, ACTA2, ITGB4	ACTB, CAV1, FLNB, ITGB5, TFRC

* BH corrected *p*-value < 0.05.

## Supplementary Materials

The following are available online at ’https://www.mdpi.com/2218-273X/10/12/1678/s1, Figure S1: 2D-Gel with protein spots significantly altered by addition of AlaGln addition to PD-Fluid. Figure S2: AlaGln effect on abundances of significantly altered proteins in biological processes. Table S1: Identified proteins of HUVEC exposed to PD fluid with and without AlaGln and control medium. Table S2: Identified proteins of omental arterioles of pediatric PD patient and healthy controls. Table S3: Significantly altered proteins of HUVEC exposed to PD fluid versus control medium. Table S4: Significantly altered proteins of omental arterioles of PD patients versus healthy controls. Table S5: Pathway analysis results of proteins only identified in HUVEC. Table S6: Pathway analysis results of proteins only identified in arterioles. Table S7: Pathway analysis results of HUVEC exposed to PD fluid with AlaGln versus PD fluid. Table S8: 2D-DiGE protein identifications. Table S9: GO term enrichments in HUVEC exposed to PD fluid vs. control medium. Table S10: GO term enrichments in HUVEC exposed to PD fluid with AlaGln vs. control medium. Table S11: Proteins significantly altered by PD fluid with AlaGln annotated within the six main biological process clusters perturbed by PD fluid.
